# A unique dataset of immunological and bacteriological test results from experimental infection of European badgers *(Meles meles)* with *Mycobacterium bovis*

**DOI:** 10.1016/j.dib.2026.113029

**Published:** 2026-07-02

**Authors:** Emmanuel Belchior, Colin P.D. Birch, Roland Ashford, Dipesh Davé, Sonya Middleton, Paul Anderson, Si Palmer, Gareth A. Williams, Stephen Powell, Mark A. Chambers, Eamonn Gormley, Ana Balseiro, Marta Barral, Benoit Durand, Laetitia Canini, Sandrine Lesellier

**Affiliations:** a Anses, EPIMIM, Laboratoire de Santé Animale, Ecole Nationale Vétérinaire d'Alfort, 94701 Maisons-Alfort, France; b French Ministry of Agriculture and Food, 75007 Paris, France; c Animal and Plant Health Agency, Woodham Lane, New Haw, Addlestone, Surrey KT15 3NB, United Kingdom; d School of Veterinary Medicine, University College Dublin, Belfield, Dublin 4, Ireland; e Animal Health Department, University of León, Campus de Vegazana s/n 24071, Instituto de Ganadería de Montaña (IGM, CSIC-ULE), 24346 Grulleros, León, Spain; f Animal Science Department, NEIKER-Basque Institute for Agricultural Research and Development, Basque Research and Technology Alliance (BRTA), Derio, Spain; g Anses, Laboratoire de la Rage et de la Faune Sauvage de Nancy (LRFSN), Technopole Agricole et Vétérinaire, Domaine de Pixerécourt-Bât. H., CS 40009-54220 Malzéville, France

**Keywords:** Tuberculosis, BCG, Vaccination, Wildlife, Immunology

## Abstract

In the United Kingdom (UK) and the Republic of Ireland (ROI), European badgers (*Meles meles*) are recognised as a reservoir host of *Mycobacterium bovis (M. bovis)*, which they can transmit to cattle. Badgers are also suspected to contribute to the maintenance of *Mycobacterium bovis* in other European countries, including Spain and France. Therefore, badger vaccination can be used as a tool for the prevention and control of *M. bovis* infection in cattle.

We present individual-level immunological and bacteriological data collected between 2002 and 2020 from 374 individual captive badgers from the UK, ROI and Spain. The data were generated through standardised experimental protocols developed for badgers and optimised in the ROI (experimental challenge protocol) and at the Animal and Plant Health Agency (APHA, UK) (immunological protocols). The analysis aimed to measure antigen-specific T-cell responses and antibody responses in BCG vaccinated and non-vaccinated badgers before and after experimental infection (challenge) with live bacteria *M. bovis*.

The data were generated from individual badgers repeatedly sampled between seven and 16 times every two-to-three weeks. The data are blood-based immunological assays and bacterial culture results of clinical samples. The dataset also includes husbandry information (sex, original social group, housing pen), physiological measurements (temperature and weight), vaccine details (type, formulation, route, dose and strain) and *M. bovis* challenge parameters (dose concentration).

Specifications Table

**Table:** 

Subject	Biology
Specific subject area	*Immunological and bacteriological test results from experimentally infected European badgers (*Meles meles*) with* Mycobacterium bovis *from 2002 to 2020 in three European countries.*
Type of data	One Excel^TM^ file containing seven sheets: two sheets containing data and five sheets containing tables describing the variables of datasets Tables (format .xlsx).
Data collection	Repeated data were obtained from analysis of blood and clinical samples in anesthetised captive European badgers experimentally infected with *M. bovis*. Blood samples were analysed using whole blood, Peripheral Blood Mononuclear Cells (PBMC) or serum assays with *M. bovis*. Clinical samples were submitted to bacterial culture. Physiological data (weight, temperature) were recorded using standard veterinary equipment*.*
Data source location	Animal and Plant Health Agency, Woodham Lane, New Haw, Addlestone, Surrey KT15 3NB, UK School of Veterinary Medicine, University College Dublin, Dublin, Ireland Universidad de León, Leon, Spain and NEIKER-Basque Institute for Agricultural Research and Development, Basque Research and Technology Alliance (BRTA), Derio, Spain*.*
Data accessibility	Repository name: Zenodo Data identification number: https://doi.org/10.5281/zenodo.19660731 Direct URL to data: https://zenodo.org/records/19660731
Related research article	None

## 1 Value of the Data

• These datasets originate from a unique series of observations of captive badgers which were trapped in the wild in TB-free areas, and kept under Level 3 containment for at least 25 weeks. They collate multicentric investigations across UK, ROI and Spain reflecting substantial financial and human resources devoted to assessing vaccine efficacy in a wildlife species.
• These datasets provide a unique, standardised collection of consistent, repeated immunological and clinical measurements, enabling the kinetic and dynamic analysis of immune biomarkers. These datasets can support comprehensive modelling approaches that incorporate multiple covariates.
• These datasets provide opportunities for comparison with immunological and antibody responses datasets from other studies, thereby enhancing the understanding of cell-mediated immune responses of mustelids to infectious agents.
• These data serve as a resource for designing experimental challenge studies in mustelid species and to refine the interpretation of immunological and serological assays in wild mustelid populations.

## 2 Background

Tuberculosis in mammals caused by *Mycobacterium* bovis (hereafter referred to as bovine tuberculosis, TB), remains a significant global zoonotic disease and an economic threat to livestock in Europe. TB-infected European badgers (*Meles meles)* have been identified in the United Kingdom (UK), Republic of Ireland (ROI), Spain and France [1]. Badgers are a key component of a multi-host epidemiological system that hinders efforts to control TB in livestock. Vaccination of badgers has been proposed as a long-term strategy to reduce prevalence of infection and transmission to susceptible cattle. Furthermore, an understanding of the levels of immunity and protection against *M. bovis* could help develop blood tests to identify populations in which it circulates.

Between 2002 and 2020, experimental vaccine trials were conducted in the UK [[1], [2], [3], [4], [5], [6]], ROI [[7], [8], [9], [10]] and Spain [11,12] to assess immune response in badgers following vaccination and experimental challenge with *M. bovis*. These studies were conducted in accordance with defined protocols and standard operating procedures (SOPs) for capture, acclimatisation, vaccination, and repeated blood and clinical sampling. Cellular immune assays (ELISPOT, Interferon gamma release assay (IG), lymphocyte transformation assay (LTA)) and serological assays (Brock test, StatPak, DPP, P22 and IDEXX) were used to characterise immune profiles in BCG vaccinated and control, *M. bovis* challenged animals. In this report the datasets of individual-level longitudinal data from these studies have been gathered into a single standardised resource.

## 3 Data Description

**Fig. 1:**
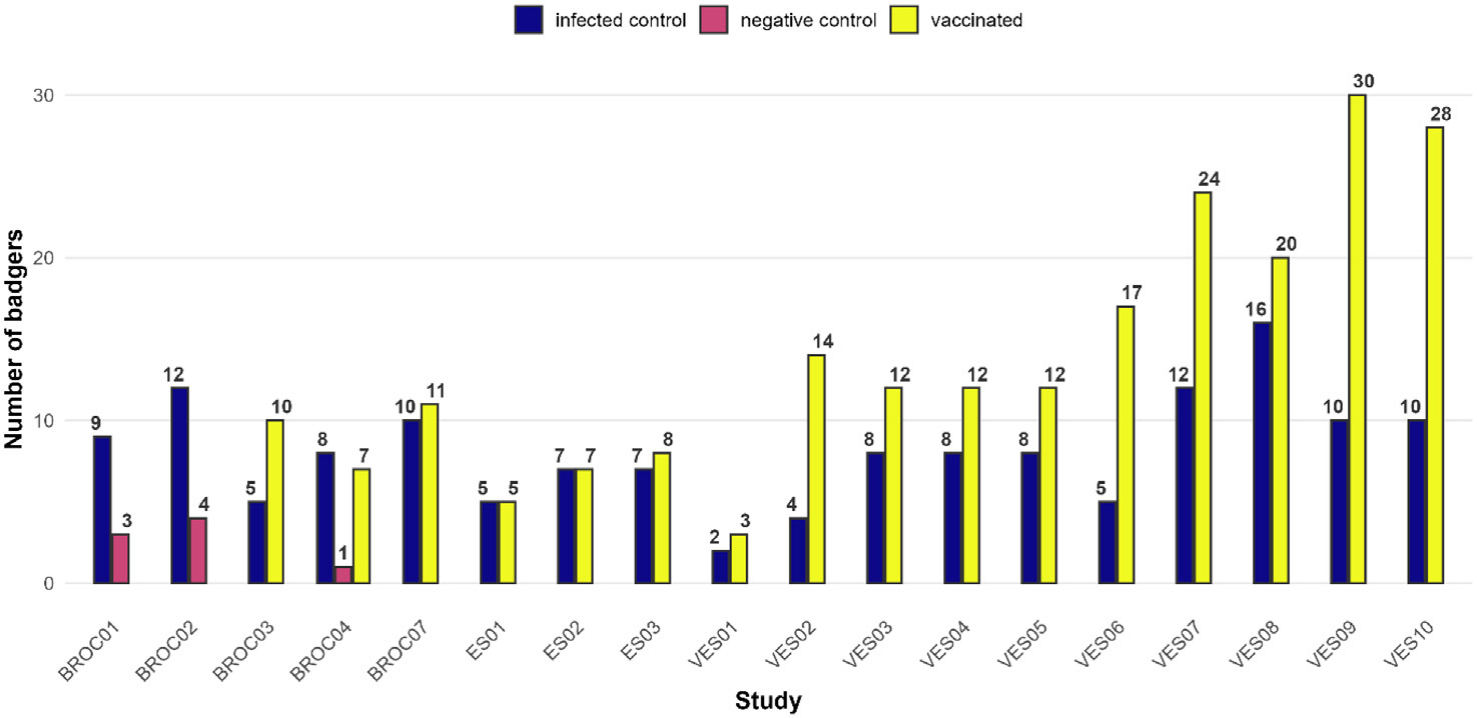
Number of badgers by study and group (negative control, infected control, vaccinated) from 2002 to 2020 in United Kingdom (VES), Republic of Ireland (BROC) and Spain (ES).

The dataset comprises 18 experiments conducted in the UK (VES1 to VES10), ROI (BROC1 to BROC4 and BROC7) and Spain (ES1 and ES2, and ES3) (Fig. 1, Table 1). In total, 374 badgers were enrolled between 18 January 2002 and 21 April 2020 (the inclusion period ranged from 2002 to 2019). Of these, 366 badgers were experimentally infected with *M. bovis* while eight badgers in three studies (BROC1, BROC2 and BROC4) were not infected (negative control). Among the 366 infected badgers, 220 were vaccinated with BCG and 146 were not vaccinated (infected control).

**Table 1:** Immunological and bacteriological test results available for challenge studies (2002–2020) in the UK (VES), Republic of Ireland (BROC), and Spain (ES), with numbers of control and vaccinated badgers.

Study	Year	Control*	Vaccinated	Total	IG ELISPOT	IGRA ELISA PBMC	IGRA ELISA WB	LTA	BROCK TEST	STATPACK	DPP	IDEXX	P22 ELISA	CULTURE
BROC1	2002	12	0	12				X	X					X
BROC2	2002	16	0	16				X						X
BROC3	2003	5	10	15				X	X					X
BROC4	2004	9	7	16	X			X	X					X
VES1	2007	2	3	5	X	X	X		X	X				X
BROC7	2007	10	11	21				X						X
VES2	2008	4	14	18	X	X	X		X	X	X			X
VES3	2009	8	12	20	X	X	X			X	X			X
VES4	2010	8	12	20	X	X	X			X				X
VES5	2011	8	12	20	X	X	X			X				X
VES6	2012	5	17	22	X	X	X			X				X
VES7	2013	12	24	36	X	X	X			X	X			X
VES8	2015	16	20	36	X	X	X				X	X		X
VES9	2016	10	30	40	X	X	X				X	X		X
ES1	2016	5	5	10	X	X	X						X	X
ES2	2017	7	7	14	X	X	X						X	X
VES10	2018	10	28	38	X	X	X				X	X		X
ES3	2019	7	8	15	X	X	X						X	X

NOTES: *control = negative control + infected control

• PBMC: Peripheral Blood Mononuclear Cells

• WB: Whole blood

• IG ELISPOT: Enzyme-linked Immunospot for badger IFN-g in PBMCs (dataset variable: ELISPOT)

• IGRA: Interferon gamma release assay specific for badgers with PBMCs or whole blood WB (dataset variables: IGPBMC or IGWB)

• ELISA: enzyme-linked immunosorbent assay (dataset variable: ELISA)

• LTA: Lymphocyte transformation assay

• DPP: Dual Path Platform test

The dataset ‘Badger_TB_Experimental_Data’ comprises seven sheets within a Microsoft Excel file (Table 2).

**Table 2:** Content of the dataset of immunological and bacteriological test results from *M. bovis* experimentally infected European badgers (*Meles meles*) from 2002 - 2020 in three European countries.

Sheets	Title	Parameters content
01	descriptionDemographic	Characteristics of badgers:
		Badger_ID, country, year, study, treatment group, sex, capture site
		Vaccination (formulation, administration, dose)
		Challenge (dose of infection, strain, challenge housing (pen))
01bis	labelsDemographic	Labels of the following variables: Pen, Vaccination formulation, Vaccination route, Capture site
02	demographicalData	1 line is a badger with all its characteristics (format 374 lines x 13 columns)
03	descriptionExperimentalData	Description (notation, measure, type, unit) and summary statistics of each individual variable
03bis	descriptionAntigens	Description of antigens used for testing
04	experimentalData	Individual badger data at each sampling time point per line (format 4,436 lines x 27 columns)
05	timelineStudies	Timelines of each study with vaccination & challenge dates

The demographic data relating to badgers are compiled in the sheet ‘02_demographicData’: identification (Badger_ID), country, study, group, sex, vaccination (formulation, route and dose of BCG vaccine), dose of *M. bovis, M. bovis* strain, capture site, pen, year.

The repeated data of sampled badgers are compiled in the sheet ‘04_experimentalData’ with the following variables:

• Identification and time: Badger_ID, time-point of sampling, study group (vaccinated/control),
• Cellular immune tests for the three antigens (PPDA, PPDB and CE) on Peripheral Blood Mononuclear cell (PBMCs) and Whole Blood and (WB): ELISPOT-PPDA (numeric), ELISPOT-PPDB (numeric), ELISPOT-CE (numeric), IGPBMC-PPDA (numeric), IGPBMC-PPDB (numeric), IGPBMC-CE (numeric), IGWB-PPDA (numeric), IGWB-PPDB (numeric), IGWB-CE (numeric), LTA-PPD (numeric), LTA-PPDB (numeric), LTA-CE (numeric),
• Serology tests: P22 (numeric), BT (numeric), STATPAK (0/1), DPP (numeric), IDEXX (numeric),
• Bacteriological tests: CULTURE (0/1),
• Physiological data: badger weight, rectal temperature.

The four metadata sheets (“01_descriptionDemographic”, “01bis_labelsDemographic”, “03_descriptionExperimentalData” and “03bis_descriptionAntigens”) give summary statistics on variables from the datasets (description of variable, type, labels, length, unique values, complete rate, mean, standard deviation, minimum, percentiles 25, 50, 75 and maximum, units, abbreviation).

The sheet called “05_timelineStudies” displays all the sampling dates organised by study, along with the vaccination and challenge dates.

## 4 Experimental Design, Materials and Methods

**Fig. 2:**
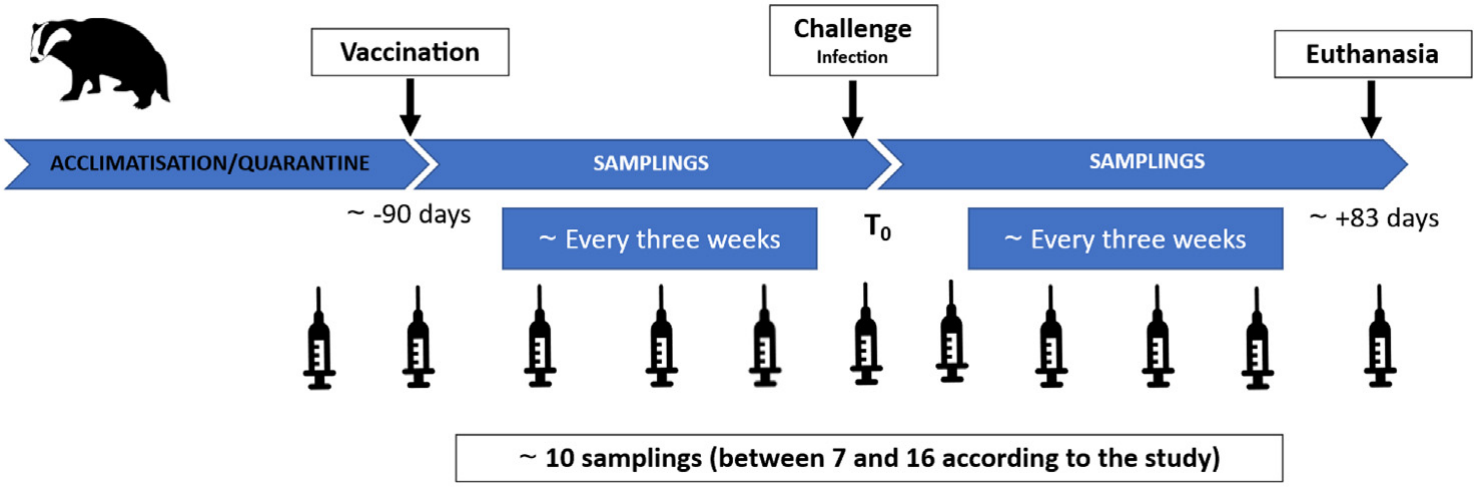
Timeline of captive studies of vaccine efficacy in badgers, from 2002 to 2019 in United Kingdom, Republic of Ireland and Spain.

All studies followed defined protocols and standard operating procedures (SOPs) (Fig. 2):

1. Capture of wild badgers from bovine tuberculosis (TB)-free areas.
2. Acclimatisation and quarantine of badgers in captivity.
3. Transfer of the TB-free badgers to open-air contained facility in ROI and to Biosafety Level 3 laboratories in UK and Spain
4. Vaccination of badgers according to assigned vaccinated/controls groups (except for BROC1 and BROC2 where no vaccination was carried out).
5. Experimental challenge of all badgers with a defined dose of *M. bovis*.
6. Collection of blood and clinical samples (tracheal aspirates, laryngeal and rectal swabs) under anaesthesia.
7. Euthanasia at the end of the trial.

### 4.1 Captive badgers

Badgers enrolled in challenge studies were trapped in TB-free areas within each country or born in captivity (eight badgers in VES7). Each badger was identified by a subcutaneous microchip. After capture, badgers were deemed TB-free based on consecutive negative IFN-γ responses (all countries) and negative culture results (only in UK). Animals were housed in stable social groups in outdoor pens (*cf.* sheet “01_descriptionDemographic”, variable “pen”) for several weeks to allow acclimatisation, vaccination and post-vaccination responses. Sizes of social groups ranged from 1 to 37 badgers, with a mean group size of 16.

Each social group was then transferred to a separate room within open-air contained facility in ROI, where handling and experimental procedures complied with Biosafety Level 3 requirements, and Biosafety Level 3 facilities in UK and Spain, for experimental challenge and monitoring the responses.

### 4.2 Vaccination and challenge

Each study (except BROC1 and BROC2) involved at least one group of badgers vaccinated with BCG or, in the Spanish studies (ES), with a heat-inactivated *M. bovis* vaccine (HIMB) and one group of non-vaccinated animals (controls). In three studies (BROC1, 2 and 4), eight badgers were not infected nor vaccinated (negative control). The BCG vaccine strains used were in-house broth-grown BCG Pasteur or pellicle-grown BCG Danish 1331 (Statens Serum Institute, Denmark).

Vaccination was delivered either under anaesthesia for manual inoculation (oral or intramuscular, Table 3), or by voluntary uptake of single baits containing the vaccine solution. The different oral vaccination procedures used varied depending on the study protocol and country (Table 3).

**Table 3:** Summary of challenge study protocols: name of the study, vaccine (type of vaccine, route of administration, dose), inoculated *M. bovis* dose, number of badgers by treatment group.

Study	Vaccine	Vaccination route	Vaccine dose (Colony Forming Units CFU/ml)	Dose of *M. bovis* (Colony Forming Units CFU/ml)	Number of badgers
BROC01	Control			10	3
				2×10^2^	3
				3×10^3^	3
	Negative control			0	3
BROC02	Control			8×10^3^	12
	Negative control			0	4
BROC03	BCG Pasteur	mucosal	5×10^5^	9×10^3^	5
		subcutaneous	5×10^5^	9×10^3^	5
	Control			9×10^3^	5
BROC04	BCG Pasteur	oral	10^8^	8×10^3^	7
	Control			8×10^3^	8
	Negative control			0	1
BROC07	BCG Danish	oral	10^8^	2×10^2^	11
	Control			2×10^2^	10
ES01	BCG Danish	oral	10^8^	10^3^	5
	Control			10^3^	5
ES02	Heat-inactivated *M. bovi*s vaccine (HIMB)	oral	10^8^	10^3^	7
	Control			10^3^	7
ES03	Heat-inactivated *M. bovis* vaccine (HIMB)	oral	5×10^7^	10^3^	8
	Control			10^3^	7
VES01	BCG Danish	intramuscular	5.4×10^6^	4.8×10^3^	3
	Control			3.7×10^3^	1
				4.8×10^3^	1
VES02	BCG Danish High Dose	intramuscular	3.2×10^6^	2.6×10^3^	4
				2.8×10^3^	2
	BCG Danish Low Dose	intramuscular	3.3×10^5^	2.6×10^3^	4
				2.8×10^3^	4
	Control			2.8×10^3^	4
VES03	BCG Danish High Dose Lipid	oral	1.86×10^8^	1.2×10^3^	8
	BCG Danish	oral	9.3×10^7^	1.2×10^3^	4
	Control			1.2×10^3^	8
VES04	BCG Danish High Dose Lipid	oral	3.2×10^8^	10^3^	4
	BCG Danish Low Dose Lipid	oral	9.65×10^6^	10^3^	8
	Control			10^3^	8
					
VES05	BCG Danish Low Dose	bait	2.9×10^7^	10^3^	6
	BCG Danish High Dose	bait	6.2×10^7^	10^3^	6
	Control			10^3^	8
VES06	BCG Danish PT	bait	10^7^	10^3^	5
	BCG Danish High Dose PT-HPO	bait	10^7^	10^3^	5
	BCG Danish Low Dose PT-HPO	bait	10^6^	10^3^	6
		oral	10^6^	10^3^	1
	Control			10^3^	5
VES07	BCG Danish High Dose	oral	4.49×10^7^	10^3^	2
		bait	4.49×10^7^	10^3^	10
	BCG Danish Low Dose	bait	1.13×10^7^	10^3^	11
		oral	1.13×10^7^	10^3^	1
	Control			10^3^	12
VES08	BCG Danish	bait	5×10^7^	10^3^	19
		oral	5×10^7^	10^3^	1
	Control			10^3^	16
VES09	BCG Danish High Dose	oral	1.2×10^9^	10^3^	10
	BCG Danish Low Dose	oral	2×10^8^	10^3^	20
	Control			10^3^	10
VES10	BCG Danish	bait	8.6×10^8^	10^3^	20
	BCG Danish	oral	8.4×10^8^	10^3^	8
	Control			10^3^	10

In the Spanish studies, oral vaccination was performed either by manual administration under general anaesthesia or by bait delivery. For manual administration, 200 µL of vaccine solution in PBS was dispensed directly onto the tonsils using disposable Pasteur pipettes. For bait delivery, the vaccine was incorporated into bait balls (3 cm diameter) composed of peanut butter, natural peanuts and oat flakes. Each bait was injected with 200 µL of HIMB vaccine, corresponding to 10^7^ inactivated CFU per bait.

In the ROI studies, badgers were vaccinated by passing a cannula approximately 10 cm down the oesophagus and inoculating 1 mL of the BCG–lipid mixture. In the study BROC03, the mucosal vaccination was administered by intranasal aerosol and conjunctival instillation.

In the English studies (VES3 and VES4), oral BCG vaccine was administered to anesthetized badgers using a syringe and catheter positioned in the proximal oesophagus. In VES9 and VES10, vaccination was performed either by direct manual administration into the oral cavity or through voluntary consumption of vaccine-containing bait. For manual administration, at least 2.0 × 10^8^ CFU of BCG were delivered directly into the oral cavity. For bait vaccination, badgers voluntarily consumed bait containing BCG, with approximately 8.6 × 10^8^ CFU incorporated into each bait in VES10. Thirteen weeks following vaccination, anaesthetised badgers were challenged experimentally by bronchial instillation (with a fiberscope targeting the right medial lung lobe) of 10 to 9000 Colony Forming Units (CFU)/millilitre of *M. bovis* suspension. The *M. bovis* strain used for challenge in UK was the spoligotype SB0140, isolated in 1997 from an infected wild badger, and in Spain spoligotype SB0339 isolated from a tuberculous wild boar. In Ireland, the *M. bovis* strain (strain M2137; spoligotype SB0142), was originally isolated in 2001 from a tuberculous badger.

Main vaccination details (dose, formulation and route of administration) and challenge protocols (dose) are described in Table 3.

### 4.3 Anaesthesia and sampling procedures

All sampling procedures required general anaesthesia, except for the delivery of oral vaccine baits, which were consumed voluntarily.

Anaesthesia involved an intramuscular injection of approximately 10 mg/kg of ketamine (Vetalar®, Pfizer Animal Health, New York, USA), 100 µg/kg of medetomidine (Domitor®, Pfizer Animal Health) and 100 µg/kg of butorphanol (Torbugesic®, Zoetis UK Ltd, Tadworth, Surrey, UK), with occasional top-ups of isoflurane in the UK and Spain.

In Ireland, this was administered by an intramuscular injection of approximately 10 mg/kg of ketamine and 100 µg/kg of medetomidine, using a thumb-controlled pole syringe (Field Development and Supply, LLC, USA).

On these occasions, the weight (measured in kilograms (kg)) and the rectal temperature (measured in Celsius degrees (°C)) of each badger were measured with calibrated scale and paediatric thermometer in UK and Spain.

At one–three-week intervals and immediately prior to vaccination and challenge, blood was collected from the jugular vein of anesthetised badgers into BD Vacutainer® tubes (Vacutainer® serum-separating tubes, or vacutainer with heparin). Tracheal mucus was collected by aspiration with a flexible urinary catheter and dispensed into Middlebrook 7H9 broth supplemented with ADC enrichment (BD, Oxford, UK). Laryngeal and rectal swabs were collected and placed into 7H9 broth and PBS, respectively. Rectal Swabs were also collected and placed into PBS. Urine was collected by manually compressing the bladder.

All the immunological (cellular and serological) tests and clinical culture undertaken in each study are listed in Table 1.

### 4.4 Cellular immunological assays

The cellular immune response of T-cells was measured following antigenic stimulation of heparinised whole blood (WB) or of Peripheral Blood Mononuclear cell (PBMC) [13].

The antigens used were:

– purified protein derivative A (*M. avium*) (PPD-A), from APHA, formerly known as the VLA (Veterinary Laboratory Agency) or Lelystadt, at 30µg/ml,
– purified protein derivative B (*M. bovis*) (PPD-B), from VLA or Lelystadt, at 30µg/ml,
– culture filtrate protein 10 protein 10 (CFP-10) and early secretory antigenic target-6 (ESAT-6), at 5 µg/ml of CFP-10 and 5 µg/ml each of ESAT-6/CFP-10 antigen cocktail, used to measure the antigen-specific responses to infection, as these antigens are not expressed at in BCG strains.

To measure the T-cell responses, three different assays were used:

• LTA: Lymphocyte transformation assay (LTA)

As previously described by *Dalley et al.* [13], cells were cultured and stimulated with tuberculin and *M. bovis* specific antigens for five days. The proliferation of peripheral blood mononuclear cells (PBMC) was measured by uptake of 3H-thymidine, monitored by beta-spectroscopy, count per minute (net cpm), *i.e*., mean cpm from antigen-stimulated wells *minus* mean cpm from negative controls (cells incubated with medium only).

• ELISPOT: Interferon-γ release assay (IGRA) Enzyme-Linked Immunospot (ELISPOT)

A badger specific direct ELISPOT assay was carried out as described [14]. Reactive T cells were counted using an AID reader and software (Autoimmun Diagnostika GmbH, Strasberg, Germany). The ELISPOT results were expressed as the net number of spot-forming units per million cells. Net values were calculated by subtracting the background values obtained with cells stimulated with medium only, from results for specific antigen, all in duplicate.

• IG Interferon-gamma (IFN-γ) Enzyme-linked immunosorbent assay (ELISA)

The badger specific interferon-gamma (IFN-γ) assay was performed as previously described [15] on whole blood (IGWB) and on PBMC (IGPBMC). Optical density (OD) was measured at 450 nm using an ELISA plate reader. The results of all samples were expressed as net optical densities (OD).

### 4.5 Serological tests

• Brock Test ELISA
  A badger specific indirect ELISA for the detection of Immunoglobulin (Ig) G to MPB83, was carried out using the published protocol [16]. Optical density (OD) was measured at 450 nm using an ELISA plate reader. The data were reported as net OD values. A positive and a negative control were included (serum from an *M. bovis* infected and naïve badger respectively).
• StatPak
  A non-badger specific rapid lateral-flow system (Chembio Diagnostic Systems Inc.) was used for the detection of IgA, IgM, and IgG antibodies against a mixture of *M. tuberculosis* complex antigens, including MPB83, ESAT-6 and CFP-10 [17]. The results were expressed as a binary positive/negative response obtained if a line for each antigen is observed.
• DPP
  A non-badger specific lateral-flow system, Dual-Path Platform (DPP®) VetTB, (Chembio Diagnostic Systems Inc.), replaced the StatPak in 2015. This assay detects non-specific IgA, IgM, and IgG antibodies and has two immuno-chromatographic test lines, separately detecting responses to MPB83 and ESAT-6/CFP-10 fusion protein [18]. Test results were expressed in Relative light units (RLU).
• Idexx
  This badger-specific indirect conjugated ELISA test (IDEXX Inc.) detects badger specific Ig against MPB83/MPB70 using a badger specific (CF2) capture antibody. The optical density (OD) was measured at 450nm on a Versamax microplate reader (Molecular Devices LLC, San Jose, CA). Test results were expressed as net OD values at 450 nm [19].
• P22 ELISA
  This badger specific indirect conjugated ELISA test was performed during trials ES1 to ES3 to detect badger specific IgG using CF2 antibody recognition of P22 cocktail following the protocol described previously [20]. P22 is an immunopurified multiprotein complex derived from bPPD and containing the proteins MPB70 and MPB83. Optical density (OD) was measured at 450 nm using an ELISA reader. Negative control serum samples from UK tuberculosis-free captive badgers were included in every plate in quadruplicate. Positive controls were obtained from UK badgers experimentally infected with *M. bovis*. Sample results were expressed as an ELISA percentage, E%, calculated using the following formula:

E% = mean sample OD / (2 × mean of negative control OD) ×100%.

### 4.6 Clinical samples for culture

All clinical samples were submitted for *M. bovis* culture for a minimum of 12 weeks at 37°C on modified Middlebrooks slopes. Data were expressed as a binary variable positive/negative [5].

## 5 Related publications

**Table:** 

Country	Year of study	Study	Year & first author of publication	DOI
Republic of Ireland	2002	BROC1	2009, Lesellier	https://doi.org/10.1016/j.vetimm.2008.09.012
Republic of Ireland	2002	BROC2	2007, Lesellier	https://doi.org/10.1016/j.vetimm.2007.11.005
Republic of Ireland	2003	BROC3	2008, Lesellier	https://doi.org/10.1016/j.vaccine.2008.10.068
Republic of Ireland	2003	BROC3	2008, Corner	https://doi.org/10.1016/j.tube.2008.03.002
Republic of Ireland	2004	BROC4	2010, Corner	https://doi.org/10.1016/j.vaccine.2010.06.120
United-Kingdom	2007	VES1	2011, Chambers	https://doi.org/10.1098/rspb.2010.1953
Republic of Ireland	2007	BROC7	Not published	
United-Kingdom	2008	VES2	2011, Lesellier	https://doi.org/10.1016/j.vaccine.2011.03.028
United-Kingdom	2009	VES3	2017, Chambers	https://doi.org/10.3389/fcimb.2017.00006
United-Kingdom	2010	VES4	2017, Chambers	https://doi.org/10.3389/fcimb.2017.00006
United-Kingdom	2011	VES5	Not published	
United-Kingdom	2012	VES6	Not published	
United-Kingdom	2013	VES7	Not published	
United-Kingdom	2015	VES8	Not published	
Spain	2016	ES01 & ES02	2020, Balseiro	https://doi.org/10.3389/fvets.2020.00041
United-Kingdom	2016	VES9	2020, Lesellier	https://doi.org/10.3390/pharmaceutics12080782
United-Kingdom	2018	VES10	2020, Lesellier	https://doi.org/10.3390/pharmaceutics12080782
Spain	2019	ES03	2023, Juste	https://doi.org/10.1016/j.heliyon.2023.e19349
United-Kingdom	2025	All studies	2025, Robertson	https://doi.org/10.1016/j.prevetmed.2025.106464

## Limitations

Immune responses were measured in healthy (even if they were infected), experimentally infected animals. This may not translate fully to natural infection of wild badgers under the influence of a multitude of confounding factors.

## Ethics Statement

All the challenge studies were authorised by national ethical bodies under the directive 2010/63/UE.

United Kingdom: all experimental procedures underwent ethical approval by the establishment and were authorised with Home Office approved endpoints referring to the welfare of badgers in captivity.

Republic of Ireland: all work with badgers was carried out under licences issued by National Parks and Wildlife Service and the Department of Health and Children and ethical approval was obtained from the UCD animal ethics committee.

Spain: the animal studies were reviewed and approved by the licensing committees from Government of Principality of Asturias and Government of Basque Country.

## CRediT Author Statement

**Emmanuel Belchior:** Data curation, Visualisation, Writing, Original draft. **Colin P. D. Birch:** Data curation, Methodology, Investigation, Formal analysis, Writing-Reviewing and Editing. **Roland Ashford:** Methodology, Resources, Investigation, Writing-Reviewing and Editing. **Dipesh Davé:** Resources, Validation. **Sonya Middleton:** Methodology, Resources, Investigation. **Paul Anderson:** Resources, Validation. **Si Palmer:** Methodology, Resources, Investigation. **Gareth A. Williams:** Methodology, Resources, Investigation. **Stephen Powell:** Software, Methodology, Resources. **Mark A. Chambers:** Conceptualisation, Data curation, Supervision, Methodology, Investigation, Writing-Reviewing and Editing. **Eamonn P. Gormley**: Conceptualisation, Resources, Data curation, Supervision, Methodology, Investigation, Writing-Reviewing and Editing. **Ana Balseiro:** Conceptualisation, Resources, Data curation, Supervision, Methodology, Investigation, Writing-Reviewing and Editing. **Marta Barral:** Data curation, Supervision, Methodology, Investigation, Writing- Reviewing and Editing. **Benoit Durand:** Supervision, Writing-Reviewing and Editing. **Laetitia Canini:** Supervision, Writing-Reviewing and Editing. **Sandrine Lesellier:** Conceptualisation, Resources, Data curation, Supervision, Methodology, Investigation, Writing-Reviewing and Editing.

## Data Availability

ZENODOBadger_TB_Experimental_Data.xlsx (Original data).
